# Polypharmacy and Psychological Distress May Be Associated in African American Adults

**DOI:** 10.3390/pharmacy7010014

**Published:** 2019-01-24

**Authors:** Shervin Assari, Mohsen Bazargan

**Affiliations:** 1 Department of Psychiatry, School of Medicine, University of Michigan, Ann Arbor, MI 48109-2029, USA; 2 Center for Research on Ethnicity, Culture and Health, School of Public Health, University of Michigan, Ann Arbor, MI 48109-2029, USA; 3 Department of Psychology, University of California Los Angeles, Los Angeles, CA 90095, USA; 4 Department of Family Medicine, University of California Los Angeles, Los Angeles, CA 90095, USA; mohsenbazargan@cdrewu.edu; 5 Department of Family Medicine, Charles R. Drew University of Medicine and Sciences, Los Angeles, CA 90095, USA

**Keywords:** race, ethnicity, Blacks, African Americans, polypharmacy, medications, psychological distress

## Abstract

**Background:** Compared to Whites, African Americans are at a higher risk of multiple chronic conditions, which places them at a higher risk of polypharmacy. Few national studies, however, have tested whether polypharmacy is associated with psychological distress—the net of socioeconomic status, health status, and stress—in African Americans. **Aims:** In a national sample of African Americans in the US, this study investigated the association between polypharmacy and psychological distress. **Methods:** The National Survey of American Life (NSAL, 2003) included 3570 African American adults who were 18 years or over. This number was composed of 2299 women and 1271 men. Polypharmacy (using ≥ 5 medications) and hyper-polypharmacy (using ≥ 10 medications) were the independent variables. Psychological distress was the dependent variable. Age, gender, socioeconomic status (education attainment, income, employment, and marital status), health care access (insurance status and usual place of care), and health status (multimorbidity and psychiatric disorders) were the covariates. Linear multivariable regression was applied to perform the data analysis. **Results:** Both polypharmacy and hyper-polypharmacy were associated with psychological distress. This association was significant after controlling for all the covariates. **Conclusions:** African Americans with polypharmacy, particularly those with hyper-polypharmacy, are experiencing higher levels of psychological distress, which itself is a known risk factor for poor adherence to medications. There is a need for a comprehensive evaluation of medications as well as screening for psychopathology in African Americans with multiple medical conditions.

## 1. Background

Although various definitions are available [1], many scholars define polypharmacy as the concomitant use of multiple medicines [2]. As polypharmacy increases the risk of drug–drug interactions and reduces adherence to the necessary medications [3,4], it is being considered as a suggestive and probable indicator of the inappropriate use of medications [5,6,7]. Due to population aging, many countries are experiencing an increase in the prevalence and burden of polypharmacy [8].

Polypharmacy is also a risk factor with major health consequences [9], such as cognitive decline and falls [3,4], which impose an enormous economic burden on patients, individuals, the health care system, and society [10]. This effect is mainly because polypharmacy reduces medication adherence and increases inappropriate drug use and adverse drug reactions [11,12]. Polypharmacy increases the risk of hospitalization, morbidity, and mortality [13,14], which is important for at least two reasons. First, drug interactions and adverse drug reactions are responsible for about 12% of all the hospitalizations of older adults, and, second, almost half of such hospital admissions are preventable [15]. The negative consequences of polypharmacy are particularly high in older adults, who are more fragile and experience age-related physiological decline [16].

Although the medical, cognitive, and economic risks of polypharmacy are well known [17], less is known about the possible link between polypharmacy and psychological well-being. In addition, of the many epidemiological studies that have been conducted on factors associated with polypharmacy [18,19,20], very few have focused on African Americans [2,21]. In addition, even less are conducted on a national sample. Studies with local samples may help with the determination of polypharmacy in specific regions; however, broader sample sizes across the country are needed to generate nationally generalizable results that can inform national policies. 

Polypharmacy is particularly common in older adults [22,23], who are more likely to be diagnosed and treated for multimorbidity (multiple chronic conditions) and psychiatric disorders [1,24,25,26,27,28]. Thus, polypharmacy is closely linked to multimorbidity [23,29]. Polypharmacy is also associated with gender [30] and socioeconomic status [31]. As a result, any studies on the link between polypharmacy and psychological distress should control for confounders such as age, gender, socioeconomic status, and chronic disease.

Epidemiological studies that investigate the factors associated with polypharmacy among African Americans allow us to identify groups of individuals and patients who are at an increased risk of potentially inappropriate medications [32,33,34]. Such knowledge is essential for the elimination of racial disparities in polypharmacy in the US. The results of such epidemiological studies can help us address the unmet health needs of the African American community, through designing and implementing evidence-based interventions and programs that target high-risk African Americans who are at particular risk of having potentially inappropriate medications. Among various aspects of the health of African Americans, polypharmacy is a neglected area [2,21,35,36].

Polypharmacy is an understudied area in individuals with a low socioeconomic status (SES), as well as in individuals from racial and ethnic minorities. Due to their increased vulnerability, combined with lower health literacy, financial difficulties, and worse access to health care, these individuals may have a higher risk of experiencing the adverse effects of polypharmacy [16]. African American individuals, in particular, have a higher risk of polypharmacy and its associated consequences [21]. African American patients are less likely to receive the most effective and simple medication regimens [37,38,39,40,41]. A lack of access to high quality care, as well as bias in the health care system limits their access to combined and simple medications, which results in the use of a higher number of older, generic medications with complex dosing regimens [2,21]. Such disparities in the type of medications may increase inappropriate medication use and reduce the medication adherence of patients [42]. 

There is very little knowledge available about the epidemiology of polypharmacy in African American adults [21,35,36]. Among the very few studies that have been conducted in this area is a recent study by Bazargan et al. [21]. The study was conducted on a local sample of underserved older African Americans adults living in south Los Angeles (n = 400). The study showed that the rate of individuals engaged in polypharmacy (≥5 medications per day) and hyper-polypharmacy (≥10 medications per day) was 75% and 30%, respectively, which was higher than national estimates [21]. The study also suggested that about 7 of 10 African American older adults are probably engaging in inappropriate drug use. In their study, the number of health-care providers (regardless of their type) was the strongest predictor of polypharmacy. That is, individuals who had multiple providers were at an increased risk of polypharmacy. However, other factors including gender, comorbidity, and potentially inappropriate medication use were also linked to polypharmacy in the sample [21]. The study did not investigate a potential link between polypharmacy and the psychological distress of African American older adults [21]. 

There is very little knowledge available about the epidemiology of polypharmacy in African American adults [21,35,36]. Among the very few studies that have been conducted in this area is a recent study by Bazargan et al. [21]. The study was conducted on a local sample of underserved older African Americans adults living in south Los Angeles (n = 400). The study showed that the rate of individuals engaged in polypharmacy (≥5 medications per day) and hyper-polypharmacy (≥10 medications per day) was 75% and 30%, respectively, which was higher than national estimates [21]. The study also suggested that about 7 of 10 African American older adults are probably engaging in inappropriate drug use. In their study, the number of health-care providers (regardless of their type) was the strongest predictor of polypharmacy. That is, individuals who had multiple providers were at an increased risk of polypharmacy. However, other factors including gender, comorbidity, and potentially inappropriate medication use were also linked to polypharmacy in the sample [21]. The study did not investigate a potential link between polypharmacy and the psychological distress of African American older adults [21]. 

### Aims

This study used data from a nationally representative study of African American adults, the National Survey of American Life (NSAL 2003) [43] to investigate the association between polypharmacy (taking ≥ 5 medications) or hyper-polypharmacy (taking ≥ 10 medications) and psychological distress in African American adults. Demographics, SES, health-care access, and health status were controlled, as they may have confounded our association of interest. 

## 2. Materials and Methods

### 2.1. Design and Setting

Building on the National Survey of American Life (NSAL 2003) data [42], this study included 3570 African American adults that were at least 18 years old. The NSAL is one of the largest national health surveys of Blacks and African Americans in the United States. The NSAL was conducted as a part of the Collaborative Psychiatric Epidemiology Surveys (CPES 2003), funded by the National Institute of Mental Health (NIMH) [42]. 

### 2.2. Ethical Approval

The University of Michigan (UM) Institutional Review Board (IRB) approved the study protocol. The NSAL sample all provided written consent. All individuals were financially compensated, regardless of their interview mode (phone vs. face-to-face). The NSAL data were re-stored and analyzed in an anonymous and confidential manner. 

### 2.3. NSAL Sample

The African American sample in the NSAL sample was selected from large cities, other urban areas, and rural areas. The NSAL used multistage national household probability sampling. The inclusion criteria were English speaker, noninstitutionalized, resident of the US, and able and willing to consent. 

### 2.4. Interviews

Interviews were performed using computer-assisted personal interviews (CAPIs), which facilitate the interview process and enhance the data quality when the survey tool is lengthy, complex, and has some skip patterns [43]. Interviews were all in English and took an average of 100 minutes to perform. The response rate of African Americans was 71%. About 82% of the NSAL interviews were conducted face-to-face. These interviews took place in participants’ homes. The remaining interviews (18%) were phone interviews.

*Race and Ethnicity.* The participants self-identified their race and ethnicity as African American, defined as Blacks with no ancestral ties to the following 13 Caribbean countries: (1) Cuba, (2) Dominican Republic, (3) Haiti, (4) The Bahamas, (5) Jamaica, (6) Trinidad and Tobago, (7) Dominica, (8) Saint Lucia, (9) Antigua and Barbuda, (10) Barbados, (11) Saint Vincent and the Grenadines, (12) Grenada, and (13) Saint Kitts and Nevis. The study was limited to the non-Hispanic Black population, thus participants were excluded if they reported both an African American and Hispanic ethnic background.

### 2.5. Survey Measures and Study Variables

*Demographic Factors.* The demographic data in this study were region, age, and gender. Region was a categorical variable (West, Northeast, Midwest, or South). Age and gender (male [reference group] and female) were treated as interval and dichotomous variables, respectively. 

*Socioeconomic Characteristics.* The socioeconomic status indicators in this study included educational attainment, household income, marital status, and employment. Educational attainment was an interval variable (years of schooling). Household income was also an interval measure, with a higher score indicating a higher income. Employment was a nominal variable with the following three categories: (1) employed (reference group), (2) unemployed, and (3) not in labor force. Marital status was treated as a categorical variable with the following three levels: (1) married (reference group), (2) divorced/separated/widowed, and (3) never married. 

*Psychological Distress.* The Kessler measure of psychological distress (K-6) [44,45] was used to measure psychological distress. The K-6 is a 6-item inventory rated on a 5-point Likert-type scale. The K-6 is a truncated version of the K-10, which was originally developed in the US to estimate the prevalence of serious mental illness. The items are mainly focused on anxiety and depression. This measure is well-accepted and validated for use in US populations, including African Americans [44,45]. 

*Multimorbidity (Multiple Chronic Medical Disease (CMC)).* In this study, multimorbidity was defined as having three or more CMCs. The number of CMCs was measured based on the self-reported history of the doctor-diagnosed CMCs. The following 14 CMCs were measured: diabetes, arthritis/rheumatism, peptic ulcers, cancer, hypertension, chronic liver disease, chronic kidney disease, stroke, asthma, other chronic lung diseases, atherosclerosis, sickle cell disease, heart disease, and glaucoma. Participants were asked whether a doctor had ever told them that they had any of the above listed conditions. Self-reported CMC measures have been shown to be valid and reliable [46]. This study treated multimorbidity as a dichotomous variable [47,48,49,50,51,52,53,54,55,56,57]. 

*Polypharmacy.* This study measured polypharmacy by asking the participants to report their medicine use over the past week. The item was, “How many different kinds of prescription medicine have you taken during the past seven days?” Participants were instructed that, “A prescription medicine is one that you can only obtain from a doctor or by giving a doctor’s written approval or prescription to a pharmacist.” They were asked to name any prescription medicine, even if it was used only once. Polypharmacy was defined as taking five or more medications [1].

*Hyper-Polypharmacy.* Using the very same measurement protocol (item, instruction, etc.), this study also measured hyper-polypharmacy, which was defined as taking ≥ 10 medications [6]. As with polypharmacy, the time frame was the previous week, and any medicine was counted even if it was taken only once.

### 2.6. Data Analysis

To adjust for the NSAL complex sampling design, Stata version 15.0 (Stata Corp., College Station, TX, USA) was used for the data analysis. The Taylor series technique was used to handle the sampling weights, which requires re-estimation of the standard errors (SE) and variance. As a result, all the percentages, means, and inferences were nationally representative. For the multivariable analysis, applied survey linear regression models were used, with sub-pop commands. In our models, polypharmacy or hyper-polypharmacy were the main independent variables, K-6 (Kessler measure of psychological distress) was the main outcome, and demographic factors (age and gender), socioeconomic status (education attainment, household income, marital status, and employment), and health (multimorbidity and major depressive disorder (MDD)) were the covariates. The regression coefficient (B), standard errors (SE), and *p*-values were reported. 

## 3. Results

### 3.1. Descriptive Statistics

The sample included 3570 African American adults who were 18 years or older. Table 1 provides a description of the demographic factors, SES, health, polypharmacy/hyper-polypharmacy, and psychological distress. As Table 1 suggests, the prevalence of polypharmacy and hyper-polypharmacy was 9.3% and 1.1% in African Americans, respectively. 

### 3.2. Association between Polypharmacy and Psychological Distress 

Table 2 shows the results of Model 1, a linear regression with polypharmacy as the independent variable, psychological distress (K-6 score) as the outcome, and confounders in the model. Based on this model, polypharmacy was associated with higher psychological distress, above and beyond demographic factors (age and gender), SES (education attainment, household income, marital status, employment), and health (multimorbidity and depression).

### 3.3. Association between Hyper-Polypharmacy and Psychological Distress 

Table 3 shows the results of Model 2, a linear regression with hyper-polypharmacy as the independent variable and psychological distress as the outcome. Based on this model, hyper-polypharmacy was associated with psychological distress, above and beyond demographic factors (age and gender), SES (education attainment, household income, marital status, and employment), and health (number of CMCs and depression).

## 4. Discussion

This study suggests higher levels of psychological distress among African American adults with polypharmacy, particularly hyper-polypharmacy—an association that is not due to demographic factors, SES, or health status. African American adults who use multiple medications, particularly those who are prescribed ≥ 10 medications, have higher psychological distress compared to those who do not use multiple medications—an association that is a net of high age, female gender, poor SES, and worse health. 

Gender was controlled, because polypharmacy [30,31] and psychological distress [58,59,60,61] are linked to gender. Some studies [30,31,62], although not all [63], have documented that polypharmacy is more prevalent in women than men. In addition, some studies have found that women are more likely than men to receive inappropriate medications [8,30,31]. Due to gendered socializations, women may be more likely to see doctors and be diagnosed with multiple chronic diseases [58]. Additionally, women might be more likely than men to seek care for their health problems [64]. With a higher awareness of their physical and mental health symptoms [65], and with better communication skills to talk about their problems with doctors, women tend to report higher multimorbidity and psychological distress [66]. Some men perceive seeking health care and disclosing emotional distress as weakness [67,68,69], which may cause some delay in disclosing their symptoms and attending health care [70]. Some studies suggest that, compared to women, men may have a higher tendency to delay using health care [71]. Medication use is also a function of gender [72]. These differences may partly explain why polypharmacy, CMCs, and psychological distress are more common in women than men. In our study, however, we found a link between polypharmacy and distress beyond the effects of gender on both polypharmacy and psychological distress. 

We documented a link between polypharmacy and distress above all confounders, including age. High age [31] is shown to be a risk factor of polypharmacy, simply because aging is a process associated with the development of multiple chronic medical conditions (multimorbidity) that need pharmacological treatment [24]. Most people in their sixties have multimorbidity [25]. As these conditions are diagnosed, a person’s risk of polypharmacy increases [26]. At the same time, older adults may report lower levels of psychological distress [73]. 

Another factor that confounds the association is SES (educational attainment). SES impacts the quantity and quality of prescriptions (i.e., risk of polypharmacy and inappropriate medications) [8,74,75], as well as distress [76]. Low SES is a risk factor for psychological distress [76], as low SES individuals face more stressors, such as financial problems, unemployment, poverty, and daily stress in their life [77]. Future research may also investigate the same patterns in a more nuanced age breakdown, particularly in older and the oldest adults. 

Although this study does not show which factors causes which outcomes, there is a need to reduce inappropriate medication use, as well as psychological distress, in African Americans. Unfortunately, it is still unknown whether or not polypharmacy is more common in African American older adults than Whites [21]. There are also not many evidence-based interventions to reduce polypharmacy in African Americans [78,79]. Systematic reviews have shown that the existing strategies are inefficient in reducing inappropriate polypharmacy [80], highlighting an emergent need to design and evaluate interventions and programs that can effectively reduce polypharmacy, as well as inappropriate polypharmacy, in high-risk populations, particularly racial and ethnic minorities [80]. Thus, the next step is to develop evidence-based protocols and interventions that offer practical solutions to reduce polypharmacy [21]. Such interventions should be aware of the positive link between polypharmacy and distress.

### 4.1. Implications

Despite the increasing trend of polypharmacy [81] and psychological problems [82] in populations, and although both impact the morbidity and mortality of the population [81,83,84], there is a need for more investment in the prevention and treatment of these common problems in African Americans. The psychological needs of African Americans who receive polypharmacy should not be discounted.

The results derived from the current study have some public health and clinical implications to promote the health of African Americans. Individuals with high age, female gender, multiple chronic medical conditions, and psychiatric conditions are at an increased risk of polypharmacy [85], and those who report polypharmacy are the same group suffering from psychological distress. Therefore, combined screening of distress and polypharmacy in older African American women with multiple medical or mental health conditions is recommended. A comprehensive intervention should address polypharmacy as well as psychological distress.

### 4.2. Limitations

Our study had some limitations. First, given the cross-sectional design, this study cannot draw any causal inferences. Future research is needed to test if psychological distress causally contributes to inappropriate medication use. Using studies with interventional designs, we need to know if the reduction of one will result in the improvement of the other. Second, we only measured polypharmacy, as we are not aware of the nature of inappropriate medication use. In addition, polypharmacy was measured using a self-reported number of medications. There is a need to verify the responses using other sources, such as insurance data or medical charts. Third, some constructs that may confound the associations were not measured. Studies and interventions on polypharmacy and inappropriate medication use should also measure the history of psychiatric problems and somatization. Future work should go beyond counting medications and collect data on the types of medication. This will provide information on inappropriate medication use and the harmful interactions between them. Collecting data on the number of medications over the previous week was also a problem. We did not include data on caregiver status, which is particularly relevant to age (and more common in older adults) and psychological distress. 

Another limitation regarding our measurement was that medications such as aspirin for the prevention of Acute Coronary Syndrome (ACS or Cerebrovascular Accident (CVA) can either be written as a prescription or can be bought without prescription over the counter (OTC). Similarly, stool softeners, multivitamins, and many other medicines can either be prescribed or bought OTC. We also did not collect data on functional limitations, cognition, and fragility, which may be risk factors of both distress and polypharmacy. In addition, the sample was adults rather than older people. There is a need to replicate these findings in adults over age of 65. Furthermore, this study only focused on quantity of medicines, rather than quality of prescriptions. Although polypharmacy is related to inappropriate medications, there is a need to study other aspects such as inappropriate medications. This study investigated the number of prescriptions rather than inappropriate medications. The number of medications could be separate prescriptions for drugs commercially available as combination products, but due to insurance formularies may require a separate prescription for each active ingredient. Finally, the results were based on some old data that were collected in 2003. The implication of this old data may be significant for this study, as the American population is aging, and the Affordable Care Act, Medicaid expansion, and other policy changes may alter individuals’ access to health care as well as insurance. Polypharmacy could also be measured using the number of prescriptions filled, in a given time period. We need to replicate the findings using more recent surveys. Despite the above limitations, we found a link between polypharmacy and psychological distress in a nationally representative sample of African Americans for the first time.

## 5. Conclusions

To summarize, this study proposed a suggestive link between polypharmacy and psychological distress among African Americans. Given the observed overlap between the two, interventions that target polypharmacy may also consider the comorbid distress of the population, to enhance the efficacy of their interventions and address the mental and physical health needs of the population. There is a need for effective interventions that jointly evaluate inappropriate medication use, physical health, and the psychological distress of African Americans.

## Figures and Tables

**Table 1:** Descriptive results in the sample (**N** = 3570).

	***% (SE)***	***95% CI***
**Gender**		
Female	44.0 (0.01)	42.4–45.7
Male	56.0 (0.01)	54.3–57.7
**Region**		
West	16.1 (0.01)	14.2–18.3
Northeast	17.6 (0.01)	14.9–20.7
Midwest	56.6 (0.02)	52.2–61.2
South	9.5 (0.01)	7.8–11.6
**Employment**		
Employed	67.0 (0.01)	64.8–69.2
Unemployed	10.2 (0.01)	8.8–11.8
Not in Labor Force	22.8 (0.01)	20.8–24.9
**Marital Status**		
Married	41.7 (0.01)	39.6–43.9
Divorced/Separated/Widowed	26.5 (0.01)	24.9–28.2
Never Married	31.8 (0.01)	29.1–34.6
**Multimorbidity**		
No	81.9 (0.01)	80.6–83.1
Yes	18.2 (0.01)	17.0–19.4
**Depression**		
No	89.7 (0.01)	88.5–90.8
Yes	10.3 (0.01)	9.2–11.5
**Polypharmacy**		
No	90.7 (0.01)	89.6–91.7
Yes	9.3 (0.01)	8.3–10.4
**Hyper-Polypharmacy**		
No	99.0 (0.01)	98.2–99.4
Yes	1.1 (0.01)	0.61–1.8
	***Mean (SE)***	***95% CI***
**Age**	42.1 (0.53)	41.0–43.1
**Education Attainment**	12.5 (0.08)	12.3–12.6
**Income** (USD 10,000)	3.6 (0.14)	3.4–3.9
**Psychological Distress**	4.8 (0.13)	4.5–5.1

Source: The National Survey of American Life (NSAL, 2003–2004).

**Table 2:** The results of Model 1, a linear regression with polypharmacy as the independent variable and psychological distress as the outcome.

	b	95% CI
**Region**		
West	1.00	
Northeast	0.28	−0.51–1.07
Midwest	0.45	−0.47–1.37
South	−0.04	−0.85–0.77
**Gender** (Female)	0.44 *	0.06–0.82
**Age**	−0.06 ***	−0.07–0.05
**Education Attainment** (Years)	−0.24 ***	−0.32–0.16
**Income**	−0.13 **	−0.20–0.05
**Marital Status**		
Married/Partnered	1.00	
Divorced/Separated/Widowed	−0.31	−0.77–0.16
Never Married	−0.31	−0.72–0.11
**Employment**		
Employed	1.00	
Unemployed	0.89 **	0.24–1.53
Not in Labor Force	0.70 ***	0.34–1.06
**Multimorbidity**	0.91 **	0.29–1.54
**Depression**	2.60 ***	1.85–3.36
**Polypharmacy**	0.87 *	0.12–1.62
**Intercept**	9.84***	8.44–11.24

Source: The National Survey of American Life (NSAL, 2003–2004). * *p* < 0.05, ** *p* < 0.01, *** *p* < 0.001.

**Table 3:** The results of Model 2, a linear regression with hyper-polypharmacy as the independent variable and psychological distress as the outcome.

	b	% CI
**Region**		
West	1.00	
Northeast	0.26	−0.54–1.05
Midwest	0.43	−0.48–1.34
South	−0.06	−0.87–0.75
**Gender** (Female)	0.43 *	0.05–0.81
**Age**	−0.06 ***	−0.07–0.04
**Education Attainment** (Years)	−0.24 ***	−0.32–0.17
**Income**	−0.12 **	−0.20–0.05
**Marital Status**		
Married/Partnered	1.00	
Divorced/Separated/Widowed	−0.29	−0.76–0.19
Never Married	−0.29	−0.70–0.12
**Employment**		
Employed	1.00	
Unemployed	0.91 **	0.27–1.55
Not in Labor Force	0.71 ***	0.32–1.10
**Multimorbidity**	0.99 **	0.39–1.59
**Depression**	2.63 ***	1.87–3.39
**Hyper-Polypharmacy**	3.06 *	0.25–5.87
**Intercept**	9.80 ***	8.43–11.16

Source: The National Survey of American Life (NSAL, 2003–2004). * *p* < 0.05, ** *p* < 0.01, *** *p* < 0.001.
